# Crioneurolysis for the Management of Delayed Spinal Cord Injury Following Electrical Injury: A Case Report

**DOI:** 10.7759/cureus.117343

**Published:** 2026-10-02

**Authors:** Maria João Andrade-Sousa, Inês P Morais, João Constantino, Paulo Margalho, Simão Serrano

**Affiliations:** 1 Physical Medicine and Rehabilitation, Centro de Medicina de Reabilitação da Região Centro - Rovisco Pais, Tocha, PRT; 2 Physical Medicine and Rehabilitation, Unidade Local de Saúde do Médio Tejo, Leiria, PRT

**Keywords:** cryoneurolysis, electrical injury, rehabilitation, spasticity, spinal cord injury

## Abstract

Delayed spinal cord injury is a rare but clinically significant complication of high-voltage electrical trauma, and spasticity represents one of its most disabling long-term sequelae, with current pharmacological options offering only transient benefit. We report the case of a Caucasian man who sustained a high-voltage electrical injury at 43 years of age in 2001, with an entry point on the left hand and an exit point on the right foot. One year later, he developed progressive spastic paraparesis, and evaluation in 2021 revealed an incomplete paraplegia (American Spinal Injury Association Impairment Scale (AIS) grade D; neurological level, L1) with myelomalacia at D5-D7. Inpatient rehabilitation with botulinum toxin injections initially improved gait symmetry, but muscle tone progressively increased over the following two years despite escalating doses. In 2024, refractory hip adductor spasticity and pain persisted despite optimized pharmacological and physical therapy. An ultrasound-guided diagnostic bilateral block of the anterior and posterior obturator nerve branches produced marked improvement in spasticity, gait, and pain, and the patient subsequently underwent cryoneurolysis of the obturator nerve branches using a freeze-thaw-freeze protocol. At follow-up, he showed improved passive range of motion, reduced adductor tone, and improved gait capacity with a walker, allowing botulinum toxin to be redistributed to other muscle groups at lower adductor-targeted doses. Cryoneurolysis, which induces a reversible axonotmesis, offers a longer-lasting alternative to chemodenervation for refractory focal spasticity, particularly when a diagnostic nerve block confirms a clear anatomical target. This case supports its role as a valuable adjunctive option for spasticity following delayed post-electrical spinal cord injury.

## Introduction

Electrical injuries are uncommonly reported and may result in both early and delayed sequelae [1]. Early complications include amputations, fractures, visceral perforations, and cardiac dysfunction. Delayed sequelae have been reported to involve neurological complications such as cerebral, spinal cord, and peripheral nerve injuries, as well as motor neuropathies. Notably, a long latency period may intervene between the electrical injury and the onset of neurological dysfunction [2,3].

The underlying pathophysiology remains poorly understood. It has been proposed that electric current preferentially flows along structures with low resistance (e.g., muscle, nerves, and blood vessels), while causing severe heat injury in structures with high resistance (e.g., skin, ligaments, and bone), typically at the entry and exit sites [4,5]. However, any structure along the current's pathway may be affected. Early management of electrical injury has traditionally focused on fluid therapy, cardiorespiratory support, and fasciotomy when indicated, while the management of neurological complications is frequently overlooked, placing these patients at considerable risk of permanent disability [6]. The true incidence and prevalence of electrical injuries and their neurological sequelae remain difficult to establish, underscoring the need for greater clinical awareness of their neurological consequences [1].

Spinal cord injury (SCI) has been reported in sporadic cases following electrical injury, with limited literature addressing its pathophysiology, clinical course, treatment, and rehabilitation [7]. Among the complications of SCI, spasticity is particularly common, occurring in up to 80% of patients. Spasticity results from dysregulation of muscle tone and motor control and can significantly limit activity and participation, with a substantial negative impact on quality of life [6,8].

Current therapeutic options, including oral antispasmodics, botulinum toxin injections, and physical therapy, provide symptomatic relief, but each carries important limitations [9-11]. Botulinum toxin injection is widely used as first-line treatment for focal spasticity; however, its effect is transient, requiring repeat administration approximately every three to four months [7].

Cryoneurolysis, a technique originally developed for pain management, involves the controlled freezing and thawing of peripheral nerves, inducing a sustained yet reversible disruption of nerve conduction through axonotmesis. Although initially applied to target sensory nerves for pain control, there is growing interest in its potential role in the management of spasticity [12,13].

The aim of this report is to describe a case of delayed SCI as a sequela of electrical injury, with particular emphasis on the diagnostic challenges and the management of the resulting spasticity, including the use of cryoneurolysis as a therapeutic approach.

## Case presentation

We report the case of a Caucasian man who was 68 years old at the time of intervention in 2025 and had sustained a high-voltage electrical injury at 43 years of age in 2001, with an entry point on the left hand and an exit point on the right foot. At the time of the accident, he was transported to the emergency department and sustained only superficial burns; no acute neurological, cardiac, or systemic abnormalities were identified.

Approximately one year after the injury, the patient developed slowly progressive spastic paraparesis with a distal-to-proximal distribution, accompanied by muscle spasms and progressive disturbances of gait and balance. In 2021, at the age of 65, he was referred to a Spinal Cord Injury Rehabilitation consultation. He had no other relevant past medical history, apart from a recent right hip arthroplasty. Clinical examination revealed a scissoring gait pattern, midline crossing of the lower limbs during ambulation, and bilateral foot drop. Neurological examination revealed normal mental status, no meningeal signs, and intact cranial nerves. Motor examination of the International Standards for Neurological Classification of Spinal Cord Injury (ISNCSCI) lower-extremity key muscles [14] showed the following Medical Research Council (MRC) grades (right/left): L2 (hip flexors), 4/4; L3 (knee extensors), 4/4; L4 (ankle dorsiflexors), 2/2; L5 (long toe extensors), 4/4; and S1 (ankle plantar flexors), 3/3, yielding a lower extremity motor score (LEMS) of 34/50. Deep tendon reflexes were hyperreflexic, with bilateral Babinski signs and distal paresthesia of the lower limbs. No bladder or bowel dysfunction was present. These findings were consistent with incomplete paraplegia, classified as American Spinal Injury Association (ASIA) Impairment Scale (AIS) grade D, with a neurological level of injury at L1 [14]. Neuroimaging demonstrated foci of myelomalacia at the T5-T7 spinal cord levels, consistent with delayed post-electrical SCI (Figure 1).

**Figure 1:**
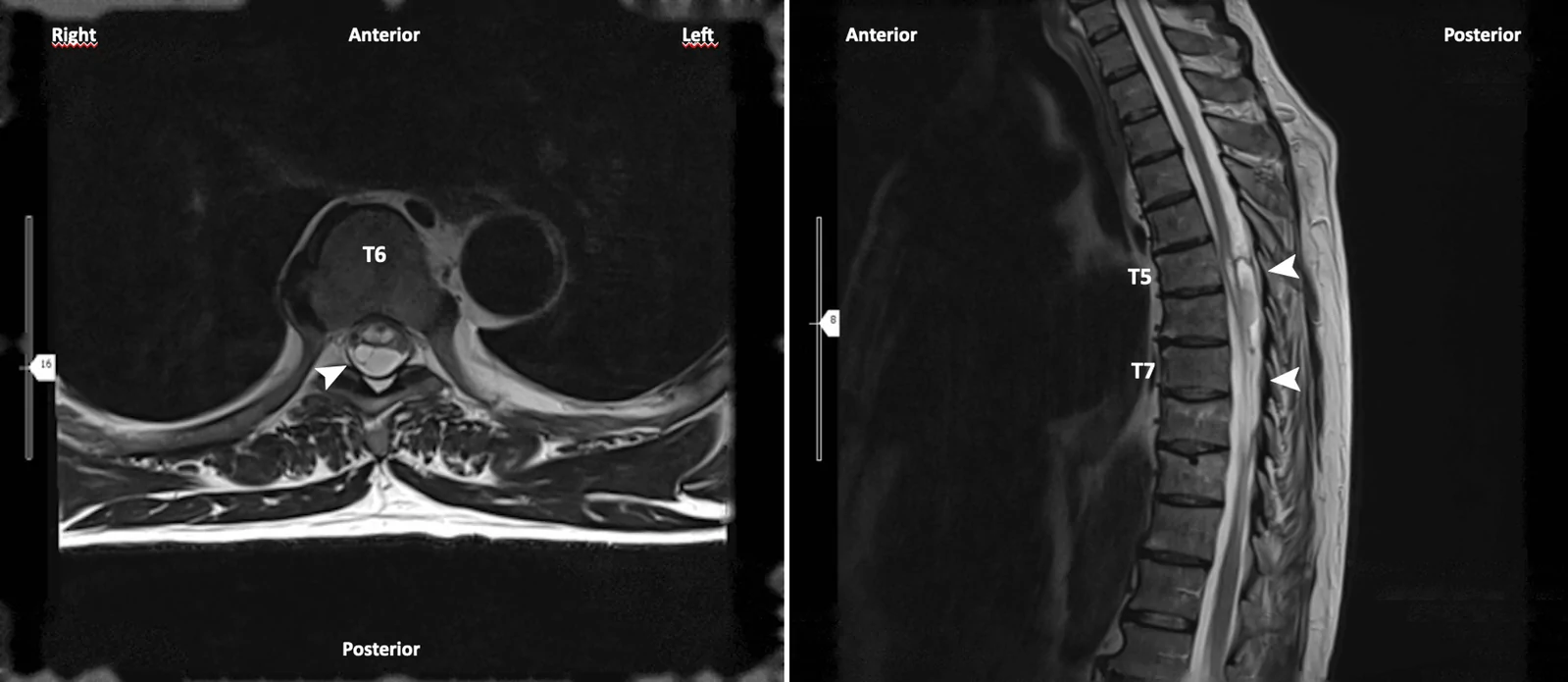
Thoracic spine magnetic resonance imaging with foci of myelomalacia at the T5-T7 spinal cord levels Thoracic spine magnetic resonance imaging, T2-weighted axial (left) and sagittal (right) sequences, showing foci of myelomalacia (T2 hyperintensity, arrowheads) at the T5-T7 spinal cord levels.

Given the functional impact of spasticity on gait and mobility, the patient was admitted between April and June 2022 to an inpatient rehabilitation program focused on functional recovery and spasticity management, including botulinum toxin (BoNT-A) injections. This intervention resulted in a significant improvement in gait symmetry. Over the following two years (2022-2024), the patient exhibited a progressive increase in muscle tone despite optimization of physical therapy, botulinum toxin injections, and oral antispastic medication.

In September 2024, the patient was readmitted due to worsening pain, restricted range of motion, and increased spasticity in the lower limbs, predominantly affecting the hip adductors (Figure 2A). Pain was bilateral, involving the thighs and lower legs, without a dermatomal or peripheral nerve distribution. It was considered of mixed origin: predominantly central neuropathic pain related to the underlying SCI, with a nociceptive component aggravated by adductor spasticity and spasms. Pain intensity was rated 6/10 on the Numeric Pain Rating Scale (NPRS). Spasticity was graded using the Modified Ashworth Scale (MAS), revealing grade 2 in the right knee flexors and extensors and grade 3 in the bilateral hip adductors. Passive range of motion was markedly limited, with right hip flexion of 45° and bilateral hip abduction of 10°. Manual muscle testing (right/left) showed grade 3/2 strength in hip flexion, hip extension, knee extension, and ankle dorsiflexion/plantarflexion. Gait capacity was significantly compromised (Table 1).

**Figure 2:**
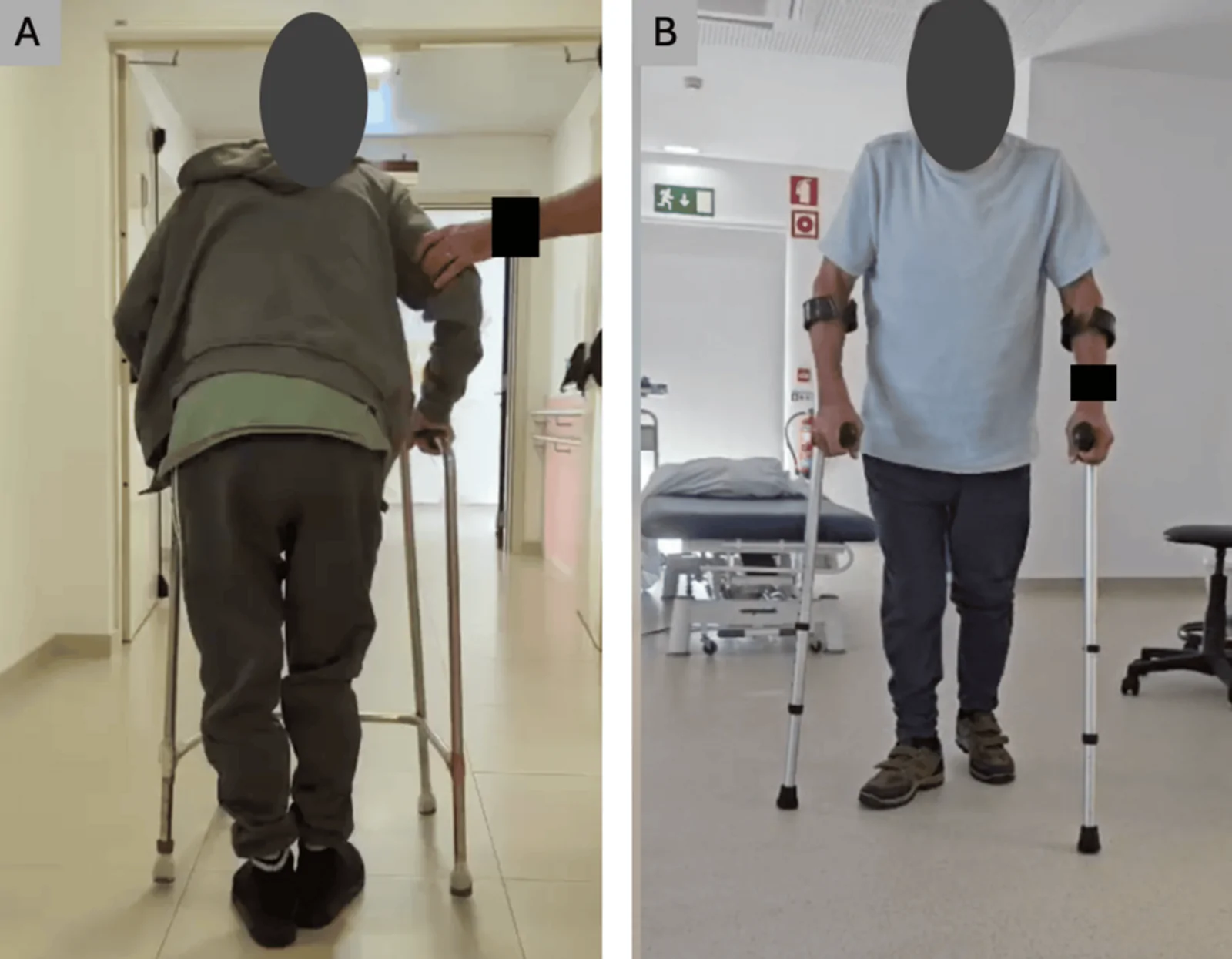
Gait before and after obturator nerve cryoneurolysis (A) September 2024: gait capacity compromised with a walker and assistance from a third person, showing a scissoring pattern with midline crossing of the lower limbs due to adductor spasticity. (B) May 2025: follow-up assessment, showing independent gait with two Lofstrand crutches, with reduced scissoring and improved stance.

**Table 1:** Clinical outcomes before (September 2024) and after (May 2025) obturator nerve cryoneurolysis NPRS: Numeric Pain Rating Scale; MAS: Modified Ashworth Scale

	Pre-procedure	Post-procedure
Pain (NPRS)	6/10	2/10
Spasticity (MAS) (right/left)	Hip adductors: 3/3; Knee extensors: 2/2; Knee flexors: 2/2	Hip adductors: 2/1; Knee extensors: 2/1; Knee flexors: 2/1
Passive range of motion	Right hip flexion: 45°; Bilateral hip abduction: 10°; Bilateral knee extension: -20°	Right hip flexion: 80°; Hip abduction: 35° (right), 45° (left); Bilateral knee extension: 10° flexion contracture

Given the refractory nature of the adductor spasticity and associated pain, an ultrasound-guided diagnostic nerve block of the anterior and posterior branches of both obturator nerves was performed, using 3 mL of 2% lidocaine at each site under direct visualization in the transverse plane. The objectives of the block were to improve joint range of motion and gait, reduce lower limb spasticity (specifically hip adduction), and decrease pain intensity. The procedure resulted in a marked improvement in the spastic pattern, gait, and pain, confirming the obturator nerve as a key contributor to the clinical presentation and supporting proceeding to a longer-acting neurolytic technique.

In March 2025, given the favorable and reproducible response to the diagnostic block, the patient underwent cryoneurolysis (CO_2_ gas) of the obturator nerve branches, with the therapeutic aim of prolonging symptom relief beyond that achieved with botulinum toxin injections or diagnostic nerve block alone. The procedure was performed percutaneously using a cryoprobe under ultrasound guidance, and electrical stimulation was used to confirm accurate targeting of the intended nerve branches. A freeze-thaw-freeze cycle was applied, consisting of three minutes of freezing at -80°C, followed by one minute of thawing and a further three minutes of freezing (Figure 3). To ensure adequate denervation of the spastic adductor musculature, two lesions were created around the nerve in cross-section, above and below, in each targeted nerve branch (anterior and posterior).

**Figure 3:**
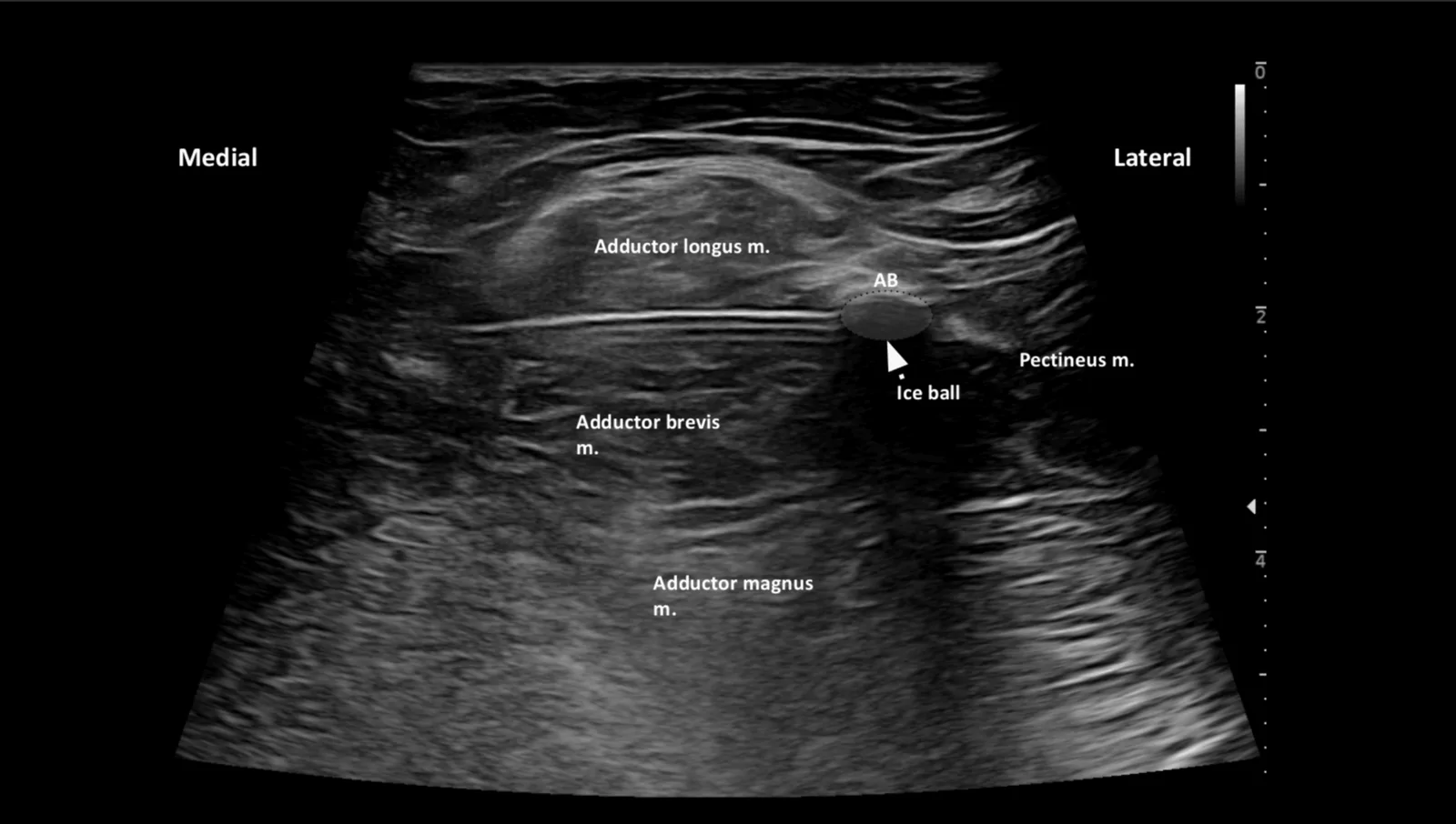
Cryoneurolysis of the anterior branch (AB) of the left obturador nerve (transverse view, medial thigh) The arrowhead indicates the ice ball at the level of the AB, seen as an anechoic area and posterior acoustic shadowing, confirming adequate freezing of the target nerve.

At follow-up in May 2025, the patient demonstrated the ability to ambulate using two Lofstrand crutches, with only mild residual difficulty with right hip abduction (Figure 2B). Passive range of motion had improved, with right hip flexion reaching 80° and hip abduction reaching 35° (right) and 45° (left); mild residual limitations persisted in knee extension (10° flexion contracture) and ankle dorsiflexion. Muscle tone, assessed using the MAS, had decreased to grade 2/1 in knee flexors, grade 2/1 in knee extensors, and grade 2/1 in bilateral hip adductors (Table 1). Adjunctive botulinum toxin injections were administered to the right gracilis, semitendinosus, semimembranosus, and rectus femoris, and to the left rectus femoris, to further optimize the functional outcome.

## Discussion

Electrical injuries are a complex and potentially life-threatening form of trauma often associated with high morbidity and mortality; the overall mortality may reach 5% [15]. Given the nature of these injuries, most of the literature has been reported as case studies, with limited data regarding the evaluation and treatment of neurological complications.

Several pathophysiological mechanisms of injury to the nervous system have been proposed, including thermal injury, electroporation, and vascular damage through direct as well as indirect injury. It is known that electrical current flows from areas of low resistance, which include muscle, nerve, and blood vessels, toward areas of high resistance, such as skin, connective tissue, and bone, which suffer greater heat injury, typically at the entry and exit sites, although damage can occur in any structure along the path of the current [4,5]. Nervous tissue provides a low-resistance route for electrical current. As the current travels through tissue, neurons with a larger surface area are more likely to be damaged by electroporation, a process in which increased cellular permeability and conductivity, caused by a permanent conformational change to membrane proteins, ultimately leads to cell death. When exposed to heat injury, vascular endothelial cells become fragile and readily undergo degenerative changes, producing injury to the vascular intima and adventitia as well as ischemic injury to the surrounding tissue. These alterations may lead to thrombosis, either secondary to endothelial injury or to diffuse degenerative change and necrosis of the vascular wall. Injury to the motor nerves innervating the blood vessels is an additional etiologic factor for vasospasm, and the resulting slow and irregular blood flow facilitates thrombus formation in both veins and arterial branches [6,16].

In 1986, Varghese et al. classified the clinical manifestations of SCI following electrical trauma into immediate and delayed types [17]. Immediate SCI occurs transiently, right after the injury, and is characterized by altered mental status, respiratory arrest, and motor paralysis, usually recovering within 24 hours. In contrast, delayed SCI typically manifests as an incomplete spinal cord lesion that occurs days to months and, less commonly, years after the initial event, as in the present case [2,3]. The clinical course is characteristically marked by progressive motor dysfunction, often with relative preservation of sensory and sphincteric function [17]. Ko et al. reviewed the medical records of 13 patients with SCI due to electrical burns; no complete loss of sensation was noted, most patients initially presented with hypotonia between days 2 and 10, and all were characterized by ascending paralysis [6].

The pathophysiology of delayed post-electrical myelopathy is thought to be primarily vascular rather than the result of direct thermal or electrical injury to neural tissue; however, this mechanism has not been directly demonstrated in individual cases and remains inferred from the anatomical and clinical pattern reported across the existing literature, rather than from angiographic, diffusion-weighted, or histopathological confirmation of vascular injury. The anterior two-thirds of the spinal cord, including the lateral corticospinal tracts, the spinothalamic tracts, and the anterior horn cells together with the central gray matter, supplied only by the terminal sulcal branch of the anterior spinal artery, has been proposed as particularly susceptible to ischemic injury, with the mid-thoracic spinal cord (T4-T8) representing a watershed region especially vulnerable due to comparatively poor collateral circulation. This vascular distribution has been suggested to account for the predominantly motor deficits observed in this and other reported cases, with relative sparing of sensory pathways. In the present patient, no spinal angiography or acute-phase diffusion-weighted imaging was performed to directly demonstrate vascular compromise; the only imaging finding available was chronic myelomalacia at D5-D7 on conventional MRI, a nonspecific marker of cord tissue loss that is compatible with, but does not in itself confirm, an ischemic etiology. Nonetheless, the anatomical level of injury, together with the incomplete, motor-predominant clinical presentation (AIS D, MRC grade 4/5, hyperreflexia, bilateral Babinski sign, and preserved bladder and bowel function) and the approximately one-year latency between the electrical injury and symptom onset, is consistent with the pattern described in previous reports attributed to this vascular mechanism, even though direct evidence of vascular pathology in this specific patient is lacking [6,16].

Complete neurological recovery in delayed post-electrical SCI is rarely achieved, and most patients are left with some degree of permanent motor impairment [17]. This reinforces the importance of directing long-term management toward optimizing function and quality of life, particularly through the treatment of spasticity, one of the most disabling secondary complications of SCI, rather than expecting reversal of the underlying cord damage.

Spasticity management in this population remains a therapeutic challenge. Oral antispastic agents and physical therapy provide only partial relief, and each carries important limitations [10,11]. Botulinum toxin injections, although considered first-line therapy for focal spasticity, provide only temporary relief, and repeated treatment cycles are frequently associated with progressive dose escalation, reflecting the well-recognized transient nature of chemodenervation [9]. These limitations support the search for adjunctive or longer-acting techniques in patients with refractory focal spasticity, particularly when a specific peripheral nerve can be identified as the principal contributor to the spastic pattern.

In this context, the diagnostic block of the obturator nerve served a dual purpose: confirming this nerve as the key driver of the adductor spasticity and hip pain, and predicting the response to a subsequent, longer-acting neurolytic technique, an approach with established precedent in the targeted management of hip adductor spasticity and obturator neuralgia [18]. The marked improvement in spastic pattern, gait, and pain observed with the diagnostic block supported proceeding with a neurolytic technique.

Phenol neurolysis of the obturator nerve is a long-established alternative for the management of refractory hip adductor spasticity, producing a neurolytic effect through non-selective protein denaturation that can last from several months up to approximately one year [19]. However, phenol can cause a non-negligible risk of dysesthesia and painful neuropathy when it diffuses to adjacent sensory branches, with dysesthesia rates of up to 15%, and a less predictable lesion extent and the potential for systemic toxicity following inadvertent intravascular injection [19,20].

Cryoneurolysis was therefore favored in this case, as it achieves sustained but reversible nerve disruption through axonotmesis, in which axonal continuity is interrupted while the endoneurium, perineurium, and epineurium remain intact, permitting gradual axonal regeneration and eventual recovery of nerve function [8,13]. This mechanism distinguishes cryoneurolysis from techniques causing neurotmesis and underlies its considerably longer duration of effect compared with chemodenervation, while preserving a favorable safety profile [8,12,13]. In this patient, cryoneurolysis of obturator nerve branches, performed using two cycles of freeze-thaw-freeze in each branch (three minutes at -80°C, one minute of thawing and a further three minutes of freezing), resulted in sustained improvement in hip range of motion, adductor tone, gait capacity, and pain at follow-up, allowing subsequent botulinum toxin treatment to be redistributed toward the remaining spastic muscle groups at comparatively lower adductor-targeted doses. The authors attributed the reduced hip abduction response to the patient's hip arthroplasty.

This case supports the use of cryoneurolysis in the management of spasticity and pain in delayed SCI after electrical injury, as an adjunctive or alternative technique for refractory focal spasticity, particularly when a diagnostic block confirms a clear anatomical target. Nonetheless, this is a single case report of a chronic and progressive condition, and not all information is available, as the patient was followed at other institutions and clinical records from that period could not be retrieved. Larger prospective studies are warranted to further define the role of cryoneurolysis in the management of spasticity of spinal origin, particularly following electrical injury.

## Conclusions

Delayed SCI following electrical trauma is an uncommon but clinically important entity, a phenomenon most commonly attributed in the literature to an ischemic mechanism within the anterior spinal artery territory, with the mid-thoracic spinal cord considered particularly vulnerable owing to its limited collateral circulation; however, this mechanism was not objectively demonstrated in the present case and remains a proposed, rather than confirmed, explanation. Complete neurological recovery is seldom achieved, underscoring the importance of therapeutic strategies directed at function and quality of life. In this case, a diagnostic obturator nerve block followed by cryoneurolysis of its branches was associated with improvement in adductor spasticity, pain, and gait capacity. Although limited by its single-case design and short follow-up, this report suggests that cryoneurolysis may be a useful adjunctive option in the management of refractory focal spasticity in patients with delayed post-electrical SCI, warranting further investigation in larger prospective studies.
